# ALTEA: A Software Tool for the Evaluation of New Biomarkers for Alzheimer’s Disease by Means of Textures Analysis on Magnetic Resonance Images

**DOI:** 10.3390/diagnostics8030047

**Published:** 2018-07-19

**Authors:** Carlos López-Gómez, Rafael Ortiz-Ramón, Enrique Mollá-Olmos, David Moratal, for the Alzheimer’s Disease Neuroimaging Initiative

**Affiliations:** 1 Center for Biomaterials and Tissue Engineering, Universitat Politècnica de València, Camí de Vera s/n, 46022 Valencia, Spain; carlogo5@etsii.upv.es (C.L.-G.); raorra@doctor.upv.es (R.O.-R.); 2 Radiology Department, Hospital Universitario de la Ribera, Alzira, 46022 Valencia, Spain; emolla@hospital-ribera.com

**Keywords:** Alzheimer’s disease, mild cognitive impairment, software, biomarkers, texture analysis, magnetic resonance imaging

## Abstract

The current criteria for diagnosing Alzheimer’s disease (AD) require the presence of relevant cognitive deficits, so the underlying neuropathological damage is important by the time the diagnosis is made. Therefore, the evaluation of new biomarkers to detect AD in its early stages has become one of the main research focuses. The purpose of the present study was to evaluate a set of texture parameters as potential biomarkers of the disease. To this end, the ALTEA (ALzheimer TExture Analyzer) software tool was created to perform 2D and 3D texture analysis on magnetic resonance images. This intuitive tool was used to analyze textures of circular and spherical regions situated in the right and left hippocampi of a cohort of 105 patients: 35 AD patients, 35 patients with early mild cognitive impairment (EMCI) and 35 cognitively normal (CN) subjects. A total of 25 statistical texture parameters derived from the histogram, the Gray-Level Co-occurrence Matrix and the Gray-Level Run-Length Matrix, were extracted from each region and analyzed statistically to study their predictive capacity. Several textural parameters were statistically significant (*p* < 0.05) when differentiating AD subjects from CN and EMCI patients, which indicates that texture analysis could help to identify the presence of AD.

## 1. Introduction

In 2011, it was estimated that 35.6 million people around the world suffered from dementia, whereas, in 2015, this figure already amounted to 46.8 million. At this rate, it is expected that this figure will almost double by 2030 and more than treble by 2050 [1,2]. Alzheimer’s Disease (AD) represents the most common type of dementia, accounting for an estimated 60 to 80 percent of cases. This neurodegenerative disorder is characterized by the presence of a progressive deterioration of the cognitive and behavioral functions, mainly in old age [3].

The diagnosis of AD remains nowadays fundamentally clinical, which means that it cannot be diagnosed until the first symptoms appear, or even later, because these early symptoms are usually associated with consequences due to aging [1]. Definitive diagnosis can only be made with histopathological confirmation of amyloid plaques and neurofibrillary tangles, usually at autopsy [4]. This is the main reason behind exploring new biomarkers that allow an early detection of AD, as patients could benefit from more efficacious treatments if the AD is diagnosed in its first stages, or even before the first symptoms appear. In the past years, many studies have focused on the analysis of mild cognitive impairment (MCI), as it is considered to be a prodromal stage of the disease or a transitional phase between normal ageing and AD, although not all patients with MCI develop AD [5,6,7]. Furthermore, several studies have proposed the existence of a preclinical stage prior to the appearance of symptoms, during which neuropathological typical changes of AD already occur [8,9,10]. This pre-symptomatic phase is still under research.

Imaging has played an important role in the study of AD over the past decades. Diagnostically, imaging has moved from a minor exclusionary role to a central position. In particular, structural magnetic resonance imaging (MRI) has gained more attention than other imaging techniques because it makes it possible to visualize in life the progressive cerebral atrophy that characterizes the neurodegenerative process of dementia, thus contributing to improving diagnostic accuracy [4,11,12]. This progressive cerebral atrophy firstly affects the medial temporal lobe [13], with the entorhinal cortex being the earliest site of atrophy, closely followed by the hippocampus, amygdala, and parahippocampal gyrus. Consequently, new biomarkers for early diagnosis of AD could be defined by processing and studying structural MRI of these brain structures [14,15].

In general, the features extracted from MRI typically used as biomarkers are related to volume and/or shape changes of specific brain structures, thus only taking into account macroscopic apparent alterations that occur when neurodegeneration has already taken place [16,17,18]. However, in recent years, texture analysis has been proved to be an excellent source of imaging biomarkers that describe properties of an image region usually imperceptible to the human eye. Texture analysis refers to the application of mathematical methods to quantify the appearance, structure and arrangement of the parts of a region within the image [19]. The features representing the texture of an image region are calculated by means of the analysis of the distribution of pixels. According to how these relationships between pixels are calculated, texture analysis methods can be classified into structural or geometrical, model-based, signal-processing and statistical methods [19]. In this study, we focused on statistical methods, which analyze the spatial distribution of the gray-level intensity values within an image region.

Texture analysis applications in medical imaging mainly encompass segmentation of specific anatomical structures or lesions and differentiation between pathological and healthy tissues. Focusing only on MRI, texture analysis has been applied to study a huge variety of diseases and lesions in different organs, but major attention has been paid to neurological applications [20,21]. Neurological disorders like brain tumors [22,23] or cerebrovascular diseases [24,25] have recently been studied by means of texture analysis. In the case of texture analysis applied to AD, several studies have tried to differentiate AD patients from cognitive normal (CN) and MCI patients using 2D or 3D texture features extracted from T1-weighted MRI and focusing on the hippocampus [26,27,28], other brain regions or structures [29,30,31], or even the whole brain [32,33,34,35]. Most of these studies proved that AD patients could be differentiated from CN and MCI patients using texture features with good accuracy (by means of statistical analyses or machine learning techniques), but the predictive capacity of the texture features was reduced when comparing CN and MCI patients. These shared results reinforce the necessity of exploring new texture biomarkers to identify image differences in the early stages of AD, when patients transition from cognitive normal to prodromal stage.

The main objective of the present study consisted of the development of a modular, intuitive and user-friendly software tool to analyze new potential biomarkers for AD through the acquisition of image parameters using 2D and 3D texture analysis on MRI. For this purpose, 3D T1-weighted MRI from three different populations were used: AD patients, MCI patients and CN subjects. The analysis was performed mainly in the hippocampal region using circular and spherical regions of interest (ROIs). Once this tool was developed, a cross-sectional study was performed through an exhaustive statistical analysis to study the predictive capacity of the possible texture biomarkers obtained.

## 2. Materials and Methods

### 2.1. Subjects and Data Acquisition

The images used in this study and the data used in the preparation of this article were obtained from the Alzheimer’s Disease Neuroimaging Initiative (ADNI) database (https://adni.loni.usc.edu) [36]. The ADNI was launched in 2003 as a public-private partnership, led by Principal Investigator Michael W. Weiner, MD. The primary goal of ADNI has been to test whether serial MRI, positron emission tomography (PET), other biological markers, and clinical and neuropsychological assessment can be combined to measure the progression of MCI and early AD. For up-to-date information, see www.adni-info.org.

In this study, we evaluated T1-weigthed MRI brain images of a total of 105 subjects from the ADNI2 database, comprising 35 AD patients, 35 CN patients and 35 MCI patients ranging from 57 to 88 years (74.9 ± 7.6, mean ± standard deviation). In the ADNI2 database, the MCI patients are subdivided in late MCI (LMCI) and early MCI (EMCI) according to the performance of the age-, sex- and education-adjusted normative mean in a standardized test for evaluating cognitive impairment [37]. In LMCI, impairment is identified using the original definition of MCI (performance of 1.5 standard deviations below the normative mean on a standardized test), whereas in EMCI, impairment is defined as performance between 1.0 and 1.5 standard deviations below the normative mean [38]. For our study, all 35 MCI patients were chosen from the EMCI group in order to take into account earlier stages of AD, closer to the pre-symptomatic phase.

The T1-weighted MRI images were acquired using 3T Siemens MRI scanners (Siemens Medical Solutions, Erlangen, Germany) with the following protocol: 3D magnetization prepared rapid gradient echo (MP-RAGE) sequence, repetition time (TR)/echo time (TE) = 2300/2.98 ms, tilt angle of 9 degrees, sagittal acquisition plane, in-plane resolution of 1 × 1 mm^2^, slice thickness of 1.2 mm, scan matrix 256 × 256 and field of view of 256 mm. The different image files were downloaded in NIfTI format, a format used for storing volumetric MRI data.

**Figure 1:**
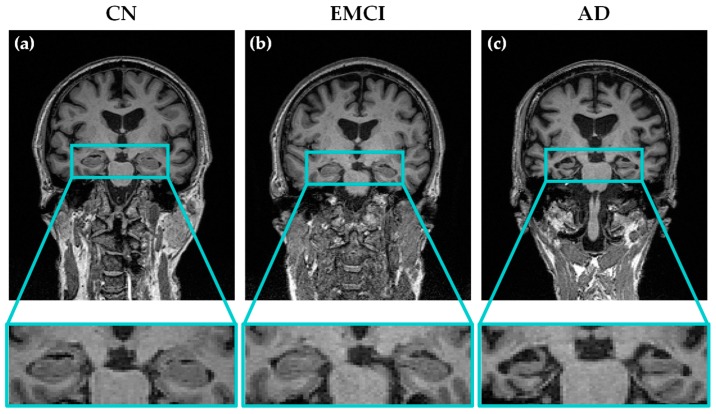
Examples of T1-weighted coronal MRI scans of three different subjects from the three groups of patients considered in this study: (**a**) cognitive normal (CN); (**b**) early mild cognitive impairment (EMCI) and (**c**) Alzheimer’s disease (AD). The progressive atrophy in the hippocampal region through the different stages of the disease can be observed.

An example of the T1-weighted MRI brain images used in our study is shown in Figure 1. The difference in the atrophy of the brain (and specifically the hippocampus) between AD, EMCI and CN patients can be seen in this figure.

### 2.2. Software

MATLAB (R2015a; The MathWorks Inc., Natick, MA, USA) was used for the development of a software tool to perform texture analysis on AD patients. MATLAB is a programming environment with its own programming language which includes data acquisition and analysis and development of applications. The user interface was created using the environment GUIDE offered by MATLAB, allowing the creation of these interfaces with the combination of graphic design through programming.

### 2.3. Regions of Interest

In the present study, we decided to evaluate the right and left hippocampi of AD, EMCI and CN patients as the regions of interest (ROIs). The segmentation of the T1-weighted MRI images was carried out on the coronal slices since this plane allows to image the hippocampus more appropriately, thus being the preferred plane for manual tracing of hippocampal borders in most of the studies [39]. Due to the difficulty of manually segmenting the hippocampus (especially in AD patients, where this structure has already been affected by atrophy), circular and spherical segmentation was performed for defining the 2D and 3D ROIs respectively. For each hippocampus (right and left), three concentric circles and spheres with different radiuses were drawn. The center of each circle or sphere was defined manually by clicking on the center of the right and left hippocampi in the coronal slice where both hippocampi showed a larger area. The radii used were of 3, 5 and 8 pixels, for both 2D and 3D segmentation. This way, the smallest ROI (ROI1, *r* = 3 pixels) contained only hippocampal tissue and the middle ROI (ROI2, *r* = 5 pixels) and biggest ROI (ROI3, *r* = 8 pixels) contained tissue from the hippocampus and from surrounding structures like the entorhinal cortex. In the end, a total of 6 ROIs were defined on each hippocampus, 3 circular ROIs (2D) and 3 spherical ROIs (3D).

Before performing texture analysis, some preprocessing techniques were applied to the image ROIs. Firstly, the image ROIs were normalized using the μ ± 3σ (µ is the mean value of the gray levels and σ is the standard deviation) to enhance the differences between groups, as proposed by Collewet et al. [40]. This method adjusts the histogram of the image ROI to µ ± 3σ by rejecting the pixels with intensities outside this range. Quantization of gray levels was also applied to the image ROIs to improve the signal-to-noise ratio of the texture outcome and to reduce the computation time of the matrix-based texture features [41]. This process refers to the reduction of levels of gray used to represent the image, which is originally represented by 65,536 gray levels (16 bits per pixel). In this case, image ROIs were quantized to 64 gray levels (6 bits per pixel).

### 2.4. Texture Analysis

Texture analysis was performed on each of the 12 preprocessed image ROIs (6 circular and 6 spherical) defined for every subject. A total of 25 statistical texture features per ROI were computed. According to the type of relationship between pixels/voxels quantified by each feature, three groups of parameters can be identified: global, local and regional [42]. The global parameters (3 features) describe the whole gray-level distribution of the image ROI, and they are obtained from the intensity histogram. The local parameters (9 features) describe the spatial relationship between pairs of pixels, and they are extracted from the Gray-Level Co-occurrence Matrix (GLCM). The regional parameters (13 features) measure the distribution of groups of connected pixels with the same gray-level values, and they are calculated from the Gray-Level Run-Length Matrix (GLRLM). Table 1 shows the list of parameters evaluated in this work.

In this study, texture features were computed with the *Radiomics* MATLAB package implemented by Vallieres et al. [43]. This package allows to compute rotation invariant GLCM and GLRLM features. Originally, GLCM and GLRLM features are dependent on direction, so different values may be obtained if the image is rotated. For texture characterization on MRI this fact is unacceptable since images from different patients may have different orientations. To solve this problem, the *Radiomics* package only computes one GLCM and GLRLM per image region by averaging the matrix values over all directions. Particularly, for 2D texture analysis, the neighboring properties of pixels in the 4 directions of the 2D space (0, 45, 90 and 135°) were averaged equally and for 3D texture analysis, the neighboring properties of voxels in the 13 directions of the 3D space were averaged differently to take into account discretization length differences [43]. Further details of the texture features evaluated in this work and how they are obtained are described in [43].

### 2.5. Statistical Analysis

A statistical study was performed to evaluate the discriminative power of each feature, that is to say, if it offered a good differentiation between the three populations considered in this work: AD, EMCI and CN. The final purpose of this analysis was to check the feasibility of these features as biomarkers of AD.

To compare the distributions of the texture parameters for the three classes, data visualization techniques and statistical tests were employed. Box-and-whiskers plots and Scatter plots were used to visualize and compare the data distribution of each class. In the case of statistical tests, each feature was evaluated with the *p*-value provided by the one-way Analysis of Variance (ANOVA) *F*-test to find statistically significant differences between the three populations, considering significant values of *p* < 0.05. However, it is important to mention that finding features with *p* < 0.05 using this test implies that there exists a significant difference between at least two of the three populations, but it is not possible to know which ones are significantly different only with this test. Therefore, additional evaluation of the difference between individual groups was needed. To this end, the Mann-Whitney-Wilcoxon (MWW) test, also called Mann-Whitney *U* test or Wilcoxon rank sum test, was used to compare the populations in pairs. This non-parametric test analog to the independent samples *t*-test does not require the normality assumption of the *t*-test, and it is recommended when the sample sizes are relatively small [44].

In statistics, when the number of statistical tests performed increases, the contrast test became more permissive, rejecting the void hypothesis more easily and increasing in this way the number of false positives by increasing the probability of randomly obtaining a significant result [44]. This problem is usually referred to as the multiple comparisons problem. To counter this effect, we decided to apply two multiple comparisons correction methods before determining which features were statistically significant. The first method applied was the Bonferroni correction, which controls the family-wise error rate. This method compensates the type I error (incorrectly rejecting the null hypothesis) and attempt to limit the probability of even one false discovery, so it is relatively strong (conservative) and, in some cases, it may lead to a very high rate of false negatives, thus increasing the type II error (accepting the null hypothesis when the alternative is true). The second method used was the Benjamini and Hochberg (BH) procedure, which controls the false discovery rate. This method attempts to control the expected proportion of false discoveries, that is, the proportion of discoveries (significant results) that are actually false positives, thus being less sensitive than the Bonferroni correction.

## 3. Results

### 3.1. ALTEA Software Tool

**Figure 2:**
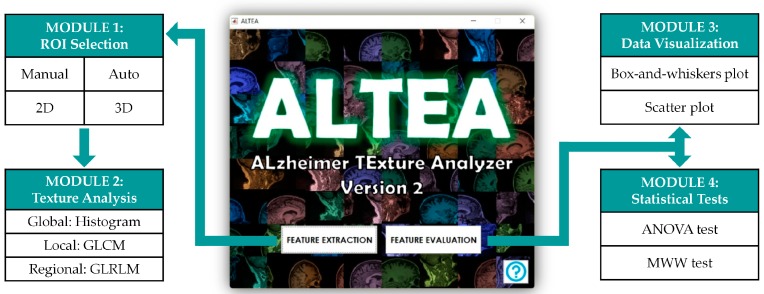
Structure of the software tool ALTEA (ALzheimer TExture Analyzer) designed for the purpose of this study. The tool has two main blocks, “Feature Extraction” and “Feature Evaluation”, and each block has two sub-blocks or modules, as shown in the image.

The first important result of this work was the creation of the software tool ALTEA (ALzheimer TExture Analyzer) specifically for this study. This software tool was developed for visualizing and segmenting MRI images, extracting different texture features and evaluating the predictive capacity of these features to check their viability as possible new biomarkers of AD. ALTEA was designed in an intuitive, user-friendly way so that any person could utilize it to perform texture analysis without major difficulties. The software is divided in two blocks: “Feature Extraction” and “Feature Evaluation” blocks. At the same time, each block is subdivided in modules, and all the modules are interconnected so the user can access the different modules in an easy and guided way. Figure 2 shows the structure of the ALTEA tool.

The “Feature Extraction” block includes the “ROI selection” and “Texture Analysis” modules. In the “ROI selection” module, the user can load the MRI image volume and define the ROIs to be analyzed (for example, the hippocampus). The MRI image is represented in three different axes using the three classic anatomical planes: axial, coronal and sagittal. ALTEA offers the option to select which plane the user wants to represent in the main axes in order to segment and define the ROIs. In addition, the use of sliding bars allows the user to navigate through the different slices in each of the three planes to search the slice for segmenting any anatomical structure. In order to select the ROIs, ALTEA offers two different options in 2D and 3D: manual segmentation and circular/spherical segmentation. The manual segmentation allows the user to manually draw the ROI on the slices and the circular/spherical segmentation allows the user to select the centers and a maximum of three radiuses to define concentric circular/spherical ROIs. Once the ROIs are defined, the user can access the “Texture Analysis” module, where the ROIs are shown and the 25 textures derived from the intensity histogram, the GLCM and the GLRLM are computed for each ROI.

The “Feature Evaluation” block includes the “Data visualization” and “Statistical tests” modules. Prior to analyzing the data, the tool asks the user to decide between 2D and 3D analysis. Once 2D or 3D data are chosen, the user can access the “Data visualization” module to visualize the distributions of the features for AD, EMCI and CN groups by means of Box-and-whiskers and Scatter plots. The user can also access the “Statistical tests” module to perform ANOVA and MWW tests and check which texture features are statistically significant when comparing populations after applying Bonferroni and BH corrections for multiple comparisons problem.

### 3.2. Results from the Statistical Analysis

The second result of this study was the analysis of the predictive capacity of the different texture features. This step was possible once the 2D and 3D texture databases had been created. The databases contained the results of extracting the 25 textures features from circular (2D) and spherical (3D) ROIs with three different radiuses (ROI1, *r* = 3 pixels; ROI2, *r* = 5 pixels; ROI3, *r* = 8 pixels) for each of the 105 subjects.

**Figure 3:**
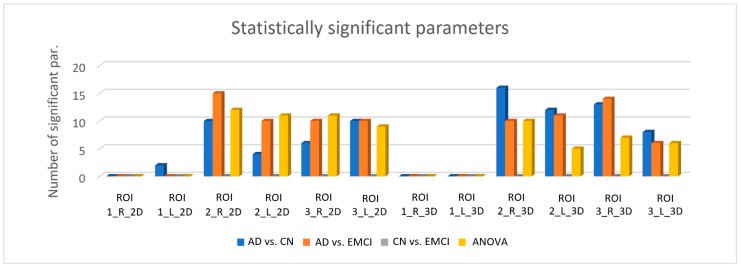
Number of values that turned out to be significant (*p* < 0.05) for each ROI with MWW and ANOVA tests and BH correction. ROI1, ROI2 and ROI3 refer to the ROIs with *r* = 3, 5 and 8 pixels respectively. The suffixes “R” and “L” correspond to those ROIs placed on the right and left hippocampus respectively. The suffixes “2D” and “3D” refer to circular and spherical ROIs respectively.

The number of statistically significant parameters (*p* < 0.05) varied notably depending on the type of ROI, as shown in Figure 3. For the six 2D ROIs (circular ROIs) the ANOVA test provided 43 significant features when applying the BH correction (16 features with the Bonferroni correction), whereas for the six 3D ROIs (spherical ROIs), the ANOVA test provided 28 significant features when applying the BH correction (15 features with the Bonferroni correction). As mentioned before, the *p*-values obtained with the ANOVA test indicated that there was a significant difference between at least two of the three population, so additional evaluation of the difference between individual groups was performed using the MWW test. Based on the results showed in Figure 3, texture parameters seem to be appropriated for differentiating AD patients from CN and EMCI patients as many parameters turned out to be significant when comparing these groups. For both 2D and 3D ROIs, the right hippocampus provided more significant parameters, and ROI2 appeared to be the best ROI regarding the size. Regarding the use of circular or spherical ROIs, the results were not very conclusive, since 3D ROIs provided more significant parameters when comparing AD and CN whereas 2D ROIs provided more significant parameters when comparing AD and EMCI in general. For both circular and spherical ROIs, none of the parameters were significant when comparing CN and EMCI and regarding the size, the parameters extracted from ROI1 had a low or null significance, since none of the texture features showed significant difference between groups when applying the Bonferroni correction. Table A1 and Table A2 in the Appendix A show the list of statistically significant features when applying the MWW test and the BH correction.

It is important to emphasize that the results showed in Figure 3 and in Table A1 and Table A2 represent the statistically significant features resulting after applying the BH correction. When applying the Bonferroni correction, the number of significant features decreased severely due to its conservative nature: for the six 2D ROIs, 8 and 12 features were significant when differentiating AD from CN and EMCI respectively, and for the six 3D ROIs, 15 and 4 features were significant when differentiating AD from CN and EMCI respectively.

Of all the parameters extracted from the circular ROIs that turned out to be statistically significant (*p*-value < 0.05), three can be outlined: Global Variance, Correlation and Autocorrelation. These parameters were statistically significant after applying BH correction for ROI2 and ROI3 and for right and left hippocampi when distinguishing AD from CN and EMCI. When applying Bonferroni correction, Global Variance gave statistically significant results for ROI2 in both hippocampi and Autocorrelation and Correlation for ROI2 in the right hippocampus.

Regarding the analysis of the spherical ROIs, the parameters that can be highlighted are Global Variance, Contrast, Correlation, Dissimilarity and Autocorrelation, with again all of them being statistically significant (*p*-value < 0.05) after applying BH correction when differentiating AD from CN and EMCI for ROI2 and ROI3 and for both hippocampi. The Correlation parameter also gave statistical significance after applying the Bonferroni correction for ROI2 in the right and left hippocampi and for ROI3 in the right one.

## 4. Discussion

Due to difficulties in accessing the brain, the diagnosis of AD is based mainly on clinical and neuropsychological tests. However, structural changes within the brain occur years before the first clinical symptoms appear and consequently the brain tissue may be damaged by the time the patient is diagnosed with AD. Therefore, there is a need to find new biomarkers of AD in its early stages and texture analysis applied on MRI may be a good approach. For this purpose, we developed the software tool ALTEA, which allows to perform 2D and 3D texture analysis on MRI in order to extract and evaluate statistically a set of texture parameters. In the present study, we used the developed tool to study the statistical significance of these texture parameters extracted from the hippocampus of T1-weighted images for differentiating between three stages of the AD: pre-symptomatic (CN), prodromal (EMCI), and advanced (AD) stages.

In the light of the obtained preliminary results, we can affirm that both 2D and 3D texture analysis are very powerful tools that could supplement and improve AD diagnosis to a great extent. A large number of the parameters obtained through texture analysis have resulted to be statistically significant to differentiate between subjects suffering from AD and subjects from the other two populations (CN and EMCI). These results indicate that texture features could be helpful for detecting AD presence. However, no statistically significant parameter was obtained to differentiate between CN and EMCI subjects; therefore, with the parameters evaluated in this study, this first stage of the illness could not be identified. The results also indicate that the size of the ROI has great relevance when analyzing textures, obtaining worse results if the ROI is too small and only includes hippocampal tissue.

Several studies have addressed the problem of identifying the presence of early AD in MRI images by studying texture analysis. Although some studies have focused on studying brain structures like the corpus callosum and thalamus [29], brain regions like white or gray matter [30,35] or even the whole brain [32], most of the analyses coincide on selecting the hippocampus as the brain structure of interest. In particular, Simões et al. proposed a method to identify and localize early-stage AD in 3D MRI volumes with a classification scheme based on Local Binary Patterns 3D patches [33] and local feature maps voxels [34]. In both studies, they observed that the patches and voxels located at the hippocampi region were highly discriminative when detecting mild AD, specifically at the left hippocampus. On the contrary, Martínez-Murcia et al. [31] applied texture analysis to 90 cortical and subcortical regions in order to differentiate AD and CN patients, and they concluded that the texture measures from the right hippocampus provided higher classification results. In our study, the right hippocampus provided more statistically significant parameters. Differences in the performance of texture analysis for both hippocampi can be explained since some studies point out that AD patients present hippocampal asymmetry, with the left hippocampus deteriorating at a higher rate, so this asymmetry may imply an early sign of the presence of AD [45,46].

When comparing our work with related similar studies that apply texture analysis to the hippocampal region in T1-weighted MRI images, our results are in accordance with those provided by these studies. In Zhang et al. [26], they studied the differentiation between 17 AD and 17 CN patients with a classification approach based on histogram, gradient, GLCM and GLRLM features extracted from spherical regions situated in the hippocampus and entorhinal cortex. They determined that too-small ROIs offered worse results than those of major size, a conclusion supported by the results obtained in the present work. Additionally, although the texture features tested by us and by them were not exactly the same, they highlighted four GLCM features (Difference entropy, Contrast, Homogeneity and Dissimilarity) as important features. In our case, for spherical ROIs, we highlighted five features as important, and four of them were also extracted from the GLCM (Contrast, Correlation, Dissimilarity and Autocorrelation), so local heterogeneity information of the hippocampal region may play an important role for characterizing AD. Although their results were very promising, we tried to go beyond their study by increasing the number of subjects per group, including EMCI patients, comparing 2D and 3D ROIs and considering rotation invariance when computing the textures.

In Li et al. [27], the hippocampi of 12 CN, 12 EMCI and 12 AD patients were segmented manually in 3D and four rotation invariant parameters (three GLCM features and one GLRLM feature) were extracted from these ROIs. By means of statistical tests, they determined that the parameters RLN and GLN extracted from the right hippocampus were statistically significant (*p* < 0.05) for distinguishing between the three populations, although they speculated that their results might be biased due to the limited sample size. Additionally, they did not apply any multiple comparisons correction the *p*-value results. In our study, we increased the number of patients and we applied Bonferroni and BH corrections to the *p*-value results in order to reduce the number of false positives, and this is probably the main reason they found differences between CN and EMCI groups and we did not, with our analysis thus being more accurate. Additionally, the number of extracted parameters in our work increased to 25, resulting in the parameters RLN and GLN being statistically significant, but not within the group of more relevant variables.

One major concern about this work is related to the ROI definition. In this study, we decided to work with circular and spherical ROIs instead of segmenting manually or automatically the whole hippocampal region. Although we implemented a manual segmentation functionality in the presented software, we decided to analyze only textures extracted from circular and spherical ROIs, because manually delineating ROIs in all patients for both hippocampi could result in a difficult, time-consuming process, not translatable to clinical practice. In future analyses, we will explore automatic segmentation techniques integrated in the MATLAB environment for segmenting brain structures (atlas-based segmentation, for example) in a fast and reliable way. This approach would be of interest because some studies report that analyzing the whole region tissue without including surrounding structures may offer better texture characterization of the tissue. However, for small regions like the hippocampus, which may present different sizes between groups of patients, geometric regions of a predefined size are recommended too. It is important to take into account that the ROI size should be sufficiently large to capture the texture information and that several texture features may be dependent on the ROI size, thus probably leading to false results due to the differences in the ROI sizes between groups [20].

Another methodological issue consisted of the lack of a spatial normalization process before defining the ROIs. As reported by Kovalev et al. [47], when working in the characterization or discrimination of small, equal-sized ROIs, spatial normalization can be omitted. Additionally, in the study of Zhang et al. [26], where they also used spherical ROIs for defining the hippocampal region, the authors stated that normalization might distort the ROIs of the MR images and destroy the texture properties of the tissue. According to these reports, we decided to keep the brain MR images in their own space and we did not register them to the standard brain for spatial normalization because our ROIs were small in comparison to the whole brain and in order to preserve the original texture properties. In our future analyses, we intend to incorporate a spatial normalization process in our software to perform a registration to the standard brain and then apply an atlas-based segmentation for extracting only the hippocampal tissue. However, before including this functionality, we will have to study how to integrate this process in our MATLAB-based software so that the segmentation of patients does not become a time-consuming process and we will have to analyze the impact of the spatial normalization process on the hippocampal ROI distortion and the texture outcome.

Our work showed other limitations or issues. Firstly, our study failed to find texture parameters that were statistically significant when comparing CN and EMCI patients, meaning that none of these parameters could accurately identify the first structural changes of the AD in the hippocampal region. Further analyses should be carried out by including a wider range of texture analysis methods (Local Binary Patterns or Wavelet and Gabor transforms, for example) or by analyzing other MRI modalities in order to look for reliable texture biomarkers for the early detection of AD. Also, more patients should be included to empower the analysis. Finally, a “Machine Learning” module is planned to be developed in order to build predictive models based on combinations of texture parameters that could diagnose accurately the AD stage of the patient under analysis.

In conclusion, our preliminary results show that, with further research, texture features could be used as biomarkers to complement the identification of the presence of AD and the specific stage of the disease in the future. The software tool developed as part of this study could be employed to perform these future studies, as it was designed to be easy-to-use for any user and it could be easily extended for performing deeper analyses in the context of AD.

## Figures and Tables

**Table 1:** List of parameters obtained per region of interest (ROI).

Texture Analysis Method	Scale	Number of Features	Feature Names
Intensity Histogram	Global	3	Global Variance
			Skewness
			Kurtosis
Gray-Level Co-occurrence Matrix (GLCM)	Local	9	Energy
			Contrast
			Entropy
			Homogeneity
			Correlation
			Sum Average
			Variance
			Dissimilarity
			Autocorrelation
Gray-Level Run-Length Matrix (GLRLM)	Regional	13	Short Run Emphasis (SRE)
			Long Run Emphasis (LRE)
			Gray-level Non-uniformity (GLN)
			Run-Length Non-uniformity (RLN)
			Run Percentage (RP)
			Low Gray-level Run Emphasis (LGRE)
			High Gray-level Run Emphasis (HGRE)
			Short Run Low Gray-level Emphasis (SRLGE)
			Short Run High Gray-level Emphasis (SRHGE)
			Long Run Low Gray-level Emphasis (LRLGE)
			Long Run High Gray-level Emphasis (LRHGE)
			Gray-level Variance (GLV)
			Run-Length Variance (RLV)

## Appendix A

**Table A1:** List of statistically significant texture parameters in the 2D texture analysis (circular ROIs) when performing the MWW test with BH correction for comparisons between individual groups.

ROI	Hippocampus	AD vs. CN	AD vs. EMCI	CN vs. EMCI
ROI1	Right	-	-	-
	Left	Sum Average, Autocorrelation	-	-
ROI2	Right	**Global Variance**, Skewness, **Correlation**, Sum Average, **Autocorrelation**, HGRE, SRHGE, LRHGE, GLV, RLV	**Global Variance**, Kurtosis, Energy, Entropy, Homogeneity, **Correlation**, **Sum Average**, **Variance**, Dissimilarity, **Autocorrelation**, **GLN**, **HGRE**, **SRHGE**, **LRHGE**, **RLV**	-
	Left	**Global Variance**, Skewness, **Correlation**, Autocorrelation	**Global Variance**, Kurtosis, Correlation, Sum Average, Variance, Autocorrelation, HGRE, SRHGE, LRHGE, RLV	-
ROI3	Right	Global Variance, **Kurtosis**, Contrast, **Correlation**, Dissimilarity, Autocorrelation	Global Variance, Kurtosis, Correlation, Sum Average, Variance, Dissimilarity, Autocorrelation, HGRE, SRHGE, LRHGE	-
	Left	Global Variance, **Kurtosis**, Correlation, Sum Average, Variance, Autocorrelation, GLN, HGRE, SRHGE, LRHGE	Global Variance, **Kurtosis**, Correlation, Variance, Autocorrelation, GLN, HGRE, SRHGE, LRHGE, RLV	-

The subset of features highlighted in bold were also statistically significant when applying the Bonferroni correction.

**Table A2:** List of statistically significant texture parameters in the 3D texture analysis (spherical ROIs) when performing the MWW test with BH correction for comparisons between individual groups.

ROI	Hippocampus	AD vs. CN	AD vs. EMCI	CN vs. EMCI
ROI1	Right	-	-	-
	Left	-	-	-
ROI2	Right	Global Variance, Kurtosis, Energy, **Contrast**, Homogeneity, **Correlation**, Sum Average, **Dissimilarity**, Autocorrelation, SRE, LRE, RLN, RP, HGRE, SRHGE, LRHGE	Global Variance, Contrast, Homogeneity, **Correlation**, Dissimilarity, Autocorrelation, SRE, LRE, RLN, RP	-
	Left	Global Variance, **Kurtosis**, Contrast, **Correlation**, Sum Average, Variance, **Autocorrelation**, Dissimilarity, GLN, HGRE, SRHGE, LRHGE	Global Variance, Skewness, Contrast, Homogeneity, **Correlation**, Dissimilarity, Autocorrelation, SRE, LRE, HGRE, SRHGE, RLN, RP	-
ROI3	Right	Global Variance, **Kurtosis**, Energy, **Contrast**, Entropy, Homogeneity, **Correlation**, Sum Average, Variance, **Dissimilarity**, **Autocorrelation**, GLN, LRHGE	Global Variance, Skewness, Kurtosis, Contrast, Homogeneity, **Correlation**, Sum Average, Dissimilarity, Autocorrelation, SRE, LRE, GLN, RLN, RP	-
	Left	Global Variance, **Kurtosis**, **Contrast**, **Correlation**, Sum Average, **Dissimilarity**, Autocorrelation, GLN	Global Variance, **Kurtosis**, Contrast, Correlation, Dissimilarity, Autocorrelation	-

The subset of features highlighted in bold were also statistically significant when applying the Bonferroni correction.
